# WikiPathways: connecting communities

**DOI:** 10.1093/nar/gkaa1024

**Published:** 2020-11-19

**Authors:** Marvin Martens, Ammar Ammar, Anders Riutta, Andra Waagmeester, Denise N Slenter, Kristina Hanspers, Ryan A. Miller, Daniela Digles, Elisson N Lopes, Friederike Ehrhart, Lauren J Dupuis, Laurent A Winckers, Susan L Coort, Egon L Willighagen, Chris T Evelo, Alexander R Pico, Martina Kutmon

**Affiliations:** Department of Bioinformatics - BiGCaT, NUTRIM, Maastricht University, 6229 ER Maastricht, the Netherlands; Department of Bioinformatics - BiGCaT, NUTRIM, Maastricht University, 6229 ER Maastricht, the Netherlands; Institute of Data Science and Biotechnology, Gladstone Institutes, San Francisco, CA 94158, USA; Micelio, 2180, Antwerp, Belgium; Department of Bioinformatics - BiGCaT, NUTRIM, Maastricht University, 6229 ER Maastricht, the Netherlands; Institute of Data Science and Biotechnology, Gladstone Institutes, San Francisco, CA 94158, USA; Department of Bioinformatics - BiGCaT, NUTRIM, Maastricht University, 6229 ER Maastricht, the Netherlands; Department of Pharmaceutical Chemistry/Pharmacoinformatics Research Group, University of Vienna, 1090 Vienna, Austria; Instituto de Ciencias Biologicas, Departamento de Bioquimica e Imunologia, Universidade Federal de Minas Gerais, Belo Horizonte, 31270-901, Brazil; Department of Bioinformatics - BiGCaT, NUTRIM, Maastricht University, 6229 ER Maastricht, the Netherlands; Department of Bioinformatics - BiGCaT, NUTRIM, Maastricht University, 6229 ER Maastricht, the Netherlands; Department of Bioinformatics - BiGCaT, NUTRIM, Maastricht University, 6229 ER Maastricht, the Netherlands; Department of Bioinformatics - BiGCaT, NUTRIM, Maastricht University, 6229 ER Maastricht, the Netherlands; Department of Bioinformatics - BiGCaT, NUTRIM, Maastricht University, 6229 ER Maastricht, the Netherlands; Department of Bioinformatics - BiGCaT, NUTRIM, Maastricht University, 6229 ER Maastricht, the Netherlands; Maastricht Centre for Systems Biology (MaCSBio), Maastricht University, 6229 EN Maastricht, the Netherlands; Institute of Data Science and Biotechnology, Gladstone Institutes, San Francisco, CA 94158, USA; Department of Bioinformatics - BiGCaT, NUTRIM, Maastricht University, 6229 ER Maastricht, the Netherlands; Maastricht Centre for Systems Biology (MaCSBio), Maastricht University, 6229 EN Maastricht, the Netherlands

## Abstract

WikiPathways (https://www.wikipathways.org) is a biological pathway database known for its collaborative nature and open science approaches. With the core idea of the scientific community developing and curating biological knowledge in pathway models, WikiPathways lowers all barriers for accessing and using its content. Increasingly more content creators, initiatives, projects and tools have started using WikiPathways. Central in this growth and increased use of WikiPathways are the various communities that focus on particular subsets of molecular pathways such as for rare diseases and lipid metabolism. Knowledge from published pathway figures helps prioritize pathway development, using optical character and named entity recognition. We show the growth of WikiPathways over the last three years, highlight the new communities and collaborations of pathway authors and curators, and describe various technologies to connect to external resources and initiatives. The road toward a sustainable, community-driven pathway database goes through integration with other resources such as Wikidata and allowing more use, curation and redistribution of WikiPathways content.

## Graphical Abstract

**Graphical Abstract:**
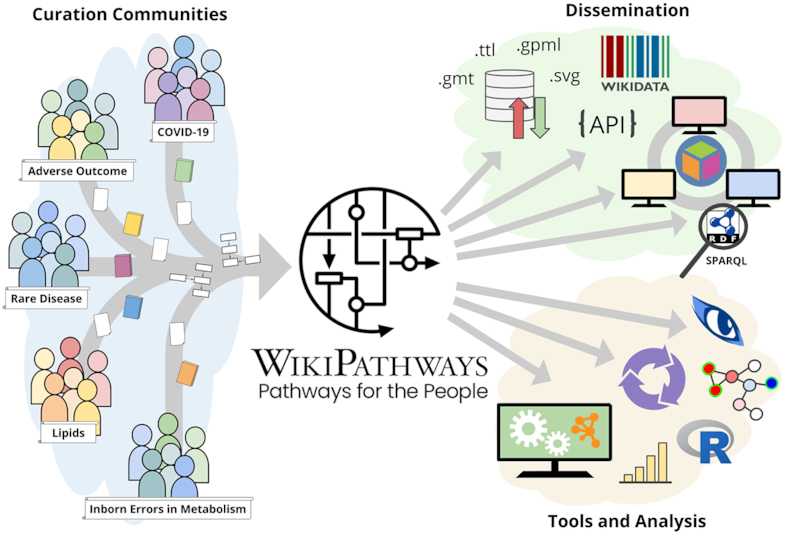
WikiPathways enables research communities to collaborate on molecular pathway curation to create reusable, machine-readable pathway models. The pathway collections are freely available and integrated with many analysis tools and resources.

## INTRODUCTION

The WikiPathways project was founded in 2007 upon the idea that everyone should be able to participate in the collection and curation of scientific knowledge (1). No single research team can match the diversity and depth of expertise represented by the greater scientific community. Putting the tools of content creators and database maintainers into the hands of content consumers completes a virtuous cycle that powers growth and quality control which scales with the acquisition of new knowledge. Our approach is reflected in open science and FAIR principles (2).

WikiPathways is a database of biological pathway models collected and curated by the research community. Anyone at any time can contribute their pathway knowledge using freely available pathway editing tools. All edits are attributed to a registered author and screened by at least one other curator by means of organized and distributed community curation. This approach allows WikiPathways to grow at the scale of new discoveries and with input from diverse sources of pathway knowledge.

As previously reported, WikiPathways relies on communities of pathway authors and curators, pathway users, and developers to assemble, update and distribute content for myriad research applications (1,3–7) In this update, we highlight unparalleled growth in content with more than seventy new pathways and thousands of revisions each year. We also present several research communities that we have collaborated with and empowered, including the COVID-19 Disease Map project (8), LIPID MAPS (9) and the rare disease community. Furthermore, to strengthen community building and curation, we started organizing monthly *Curation Cafe* events focused on selected topics, e.g. improving the quality of existing or creating novel pathways. We also detail some of the latest infrastructure updates, tool development and dissemination work which improve the free exchange of pathway information across platforms and within common analytical workflows.

## CONTENT AND GENERAL UPDATES

**Figure 1.**
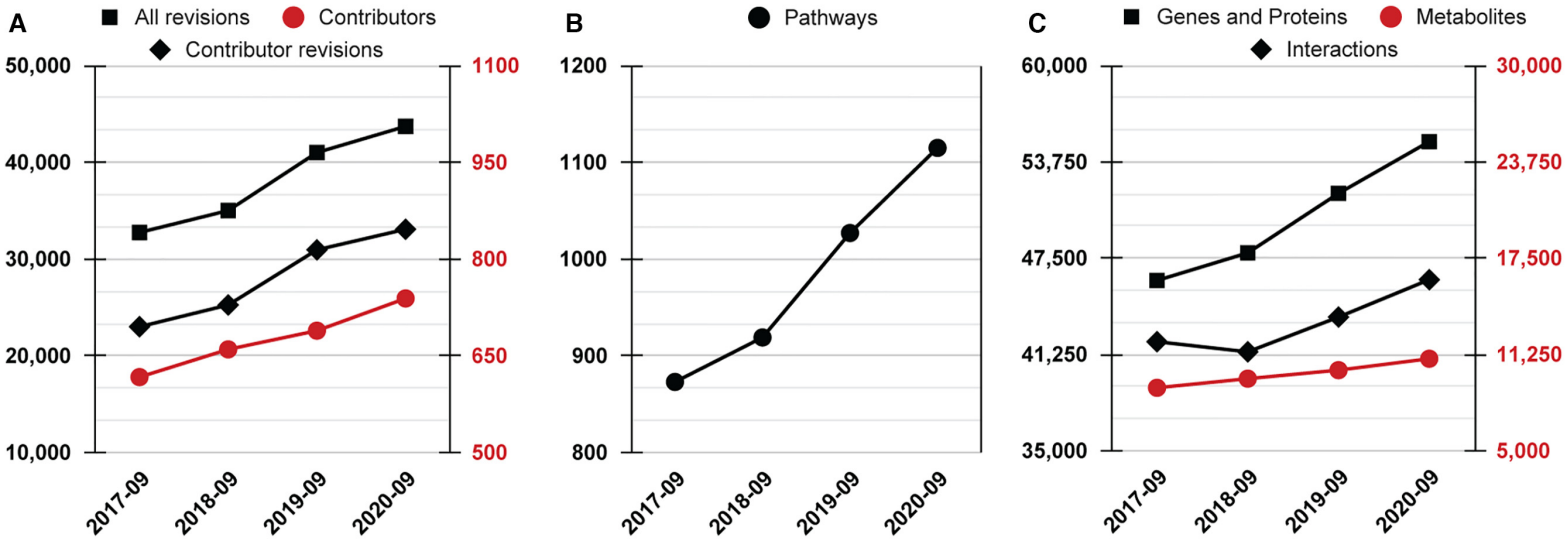
Recent growth of WikiPathways. The y-axes represent data for the human pathways accumulated in the database (approved content via http://rdf.wikipathways.org), focusing on the last three years (x-axes). (**A**) Total counts for all revisions, including contributor and automated revisions (black squares), the subset of revisions made by contributors (black diamonds) and individual contributors (red circles). (**B**) Total human pathway count. (**C**) Total counts for genes and proteins (black squares), interactions (black diamonds) and metabolites (red circles). Data colors match corresponding y-axes.

In the three years since our last update (7), over 70 new pathways per year (on average) were added to our data release (http://releases.wikipathways.org). In this section, we report on the content updates between the data releases on 10 September 2017 and 10 September 2020. Overall, WikiPathways currently contains a total of 2857 pathways for all species, out of which 1777 are included in the species-specific analysis collections. In the last three years, the content at WikiPathways has seen 10 079 user contributions, and 122 new contributors joined our community (Figure 1A). Although the content at WikiPathways represents human biology to a large extent, a total of 29 species are supported including vertebrates, invertebrates, plants, eukaryotic microorganisms and bacteria. Our human pathway collection has been extended consistently with 242 pathways (Figure 1B), and 9014 genes and proteins, of which 12% are new to the database. Furthermore, of the 1886 metabolites added, 69% were new, as a result of a concerted effort on metabolite curation. These datanodes are connected by 46 105 interactions, of which there are currently 4026 more than in September 2017 (Figure 1C).

Based on data from Google Analytics in the last three years, the main WikiPathways website has recorded on average 700 visitors per day an international audience (33% from North America, 32% from Asia, 25% from Europe). Additionally, our REST webservice API recorded 27 million requests during this three-year time span. Importantly, in an effort to produce a more accessible and sustainable resource, we regularly disseminate pathway content to third-party tools and databases (see section ‘Connections to other initiatives’), which generate secondary usage statistics not reported here.

### Pathway lifecycle

New biological knowledge is published every day, and as part of the curation process, pathway models get revised over time with this new information, in addition to other updates and corrections. Quality assurance of the WikiPathways content is accomplished continuously, by a combination of a weekly manual curation by a member of an organized team of curators, computer-assisted curation processes (7) and monthly curation cafes. Our manual curation protocol is designed as an interactive set of tasks which cover a wide range of topics, from recent edits and additions, assessing redundancy and overlap, to problematic content. It has been used successfully for the past three years and has greatly streamlined and standardized the process. The ease-of-use has also made it easy to bring in new contributors. Additionally, the computer-assisted curation tools are used effectively and its repertoire of tests has been expanded to better support the ongoing curation efforts and challenges in the WikiPathways database (https://github.com/BiGCAT-UM/WikiPathwaysCurator).

**Figure 2.**
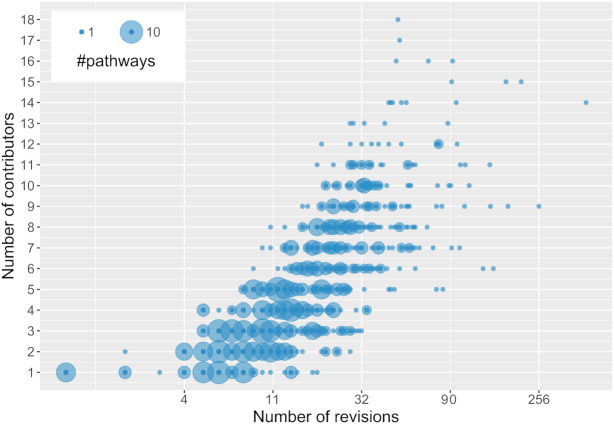
The number of revisions and contributors for all pathways in the human pathway analysis collection. The x-axis depicts how often pathways were updated (revised) on a logarithmic scale. The y-axis depicts the number of contributors who have worked on a pathway. The size of the dots expresses the number of pathways for that combination of contributors and revisions.

Each edit in a wiki-based system is recorded as a revision in the pathway history. These revisions are a measure for community activity and engagement. Pathway edits cover adding new biological knowledge, annotating the pathways with metadata (description, ontology tags), improving the layout of a pathway diagram, and any combination thereof. Figure 2 shows that pathways from the human pathway analysis collection are updated regularly. While only 6 out of 639 pathways have not been updated yet, 14 pathways have >100 revisions including the ‘Integrated Breast Cancer Pathway’ (WP1984, 445 revisions, 14 curators, (10)), the ‘Aryl Hydrocarbon Receptor’ pathway from NetPath (WP2586, 256 revisions, 9 curators, (11,12)), and the ‘Selenium Micronutrient Network’ (WP15, 208 revisions, 15 curators, (13)), displaying that the collaborative nature of WikiPathways is a clear asset to pathway curation.

### Pathway figures in published literature

Even with the continued growth at WikiPathways, approximately only 50% of protein-coding human genes are represented in pathway models. Despite the availability of free pathway modelling tools, such as PathVisio (14), CellDesigner (15) or Newt (16), the vast majority of pathway content is still shared as static images in published figures. Each month, an estimated 1000 figures representing pathway content are published and collected at PubMed Central (PMC) (17). Hence, the WikiPathways project initiated an analysis to convert published pathway figures into pathway models, using a pipeline beginning with a PMC image search, followed by machine learning, optical character recognition and named entity recognition. We identified 64 643 pathway figures published over the past 25 years and extracted 1 112 551 human genes, representing 13 464 unique genes (18). These include over 3600 genes not previously included in WikiPathways nor Reactome collections (as of January 2020). Based on enrichment analysis of disease-annotated gene sets against these pathway figures, the genes represent a wide range of diseases, including various types of cancer, cardiomyopathy, and diabetes. Prioritizing novel genes and rare diseases, we are using these published pathway figures as starting points for collaborative curation events. We have made all of the pathway figure content available via an interactive web interface (https://gladstone-bioinformatics.shinyapps.io/shiny-25years).

## PATHWAY CURATION COMMUNITIES

The collaboration with various communities is an essential part of WikiPathways, where portals serve as a functional framework for communities with focused pathway interests (http://portals.wikipathways.org). Portal maintenance instructions are provided to enable communities to design and maintain portals themselves, with assistance from the WikiPathways team if needed. Here, we highlight recent community efforts in collaboration with WikiPathways. All pathways receive a tag specifically for their community, allowing for automated downloads of these pathway collections through the REST API (http://webservice.wikipathways.org) with the ‘getCurationTagsByName’ function, and our RDF format with SPARQL queries (http://rdf.wikipathways.org, (6)).

### COVID-19

**Figure 3.**
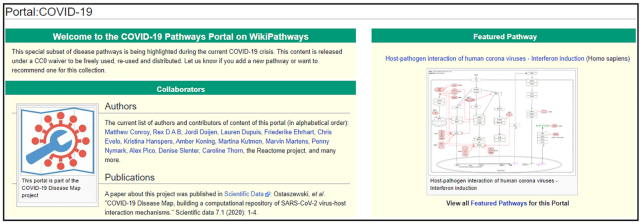
The COVID-19 Portal on WikiPathways (http://covid.wikipathways.org). The portal contains relevant information for COVID-19-related research, including all molecular pathways, contributors, and publications.

The COVID-19 Disease Map project aims to understand biological processes relevant to the COVID-19 pandemic (8). From the start of this international effort, WikiPathways has been committed to contributing to this initiative by building, curating and sharing pathway models with a liberal license (CC0) and under FAIR community standards. Currently, the WikiPathways COVID-19 portal (http://covid.wikipathways.org, see Figure 3) contains a collection of eleven molecular pathways on SARS-CoV-2 itself, nine on other coronaviruses from earlier outbreaks, and several known processes involving ACE2, the main target membrane enzyme of SARS-CoV-2 for entering host cells. Identifiers and cross-references for coronavirus genes and proteins are provided through a Wikidata project (19). Our pathway models are regularly updated and integrated into the COVID-19 Disease Map. For this initiative, we are currently adapting the data model to allow better support for multi-species pathways, annotation of evidence information and annotation of complexes.

### Rare diseases

Rare diseases affect relatively few people, with the exact definition varying between 5 and 80 individuals per 100 000 for a given rare disease. However, it is estimated that up to 5.9% of the general population is affected by a rare disease. The majority of these disorders are genetic, 4440 of 6172 in total counted by ORPHANET (20). For many disease-causing genes, there is little known about the normal gene function, and this knowledge is scattered over scientific publications and databases. Within WikiPathways there is a specialized portal for exploring, curating, and expanding the collection of rare disease pathways (http://raredisease.wikipathways.org), partnered by EJP-RD (European Joint Programme on Rare Diseases), ELIXIR (European Bioinformatics infrastructure programme) and the Dutch Rett expertise centre (Maastricht University Medical Centre). The portal is used to capture knowledge from literature and data to gain a better understanding of these complex disorders. The pathways are created and curated in collaboration with disease experts, currently covering over 60 rare diseases including very different types of diseases, e.g. laminopathies, ciliopathies, disorders of sexual development and fertility, and copy number variation syndromes.

### Inborn errors of metabolism (IEM)

Inborn errors of metabolism are a subsection of the rare disease field, which are captured in the ‘IEM portal’ (http://iem.wikipathways.org), containing molecular pathways connecting clinical biomarkers to disorders. We have started a collaboration with the authors of the book ‘Physician’s Guide to the Diagnosis, Treatment, and Follow-Up of Inherited Metabolic Diseases’ (21) and are currently processing all included pathways, as well as integrating these in the IEMbase (22). The portal currently covers 19 chapters, 23 approved pathways, over 350 diseases linked to OMIM identifiers and 68 unique Disease Ontology terms, and is expected to be expanding with the coverage of additional chapters. Examples of data analysis for these (and other) pathways can be found at https://bigcat-um.github.io/PathwayAnalysisBlauBook. The disease nodes are currently represented as Labels with hyperlinks in the pathway models. We are planning to extend the data model with a non-molecular data node (e.g. Annotation / Phenotype) that will not be used for data analysis but can be annotated with a proper identifier for computational processing of the information.

### Lipids

Lipids are a fascinating class of chemical compounds that serve several roles within organisms and are difficult to measure in a wet lab setting. The LIPID MAPS team has initiated a collaboration (9) with the WikiPathways community to maintain and extend their lipid pathway content, leading to the addition of nine highly curated lipid pathways for mouse, the original pathways are available at https://lipidmaps.org/resources/pathways/vanted.php. These pathways have been homology converted to their human counterpart and are now part of the Lipids Portal (http://lipids.wikipathways.org). Annotating individual lipids instead of lipid classes can be quite complicated; this phenomenon is in most cases due to a lack of biological knowledge on individual lipids. Furthermore, several cases are known where homology mapping between different species for proteins is hampered, e.g. for stearoyl-CoA desaturase-1 having four isoforms in mice compared to only two in humans (23).

### Adverse Outcome Pathways

Since the introduction of Adverse Outcome Pathways (AOPs) to support regulatory decision making for risk assessment of chemicals (24), the primary focus of AOP research groups has been capturing mechanistic data in written format. However, since the majority of biological processes described in AOPs are biological pathways that exist as pathway models on WikiPathways, the AOP Portal (http://aop.wikipathways.org) has been created to capture all pathways relevant to toxicological assessments (25). These molecular AOPs contribute to the understanding of AOPs and facilitate the use of various omics approaches in risk assessments (26). One challenge lies in the unique rationale behind molecular AOPs, where biological processes are connected in a chain of Key Events (KEs) that make an AOP, rather than presenting one molecular pathway. Second, KEs often describe disturbances or adverse responses already captured in molecular pathways in WikiPathways (25) and therefore AOPs are modelled as meta-pathways. These combine pathway nodes and KE nodes in one data model, which is linked to the AOP-Wiki, https://aopwiki.org).

## CONNECTIONS TO OTHER INITIATIVES

WikiPathways enables anyone to freely share, redistribute, use, and adapt pathway content in the database, by removing any barriers for people to decide to contribute to or use WikiPathways. Furthermore, WikiPathways provides a variety of options to access the data for use, through downloads in various formats for individual pathways or pathway collections, from the pathway editor and analysis software PathVisio (14), through the WikiPathways REST API and rWikiPathways R package, or through the WikiPathways SPARQL endpoint. These aspects make WikiPathways content easy to implement in services, tools, workflows or distributions.

### BridgeDb

Managing molecular pathways requires robust use of database identifiers for all pathway components (genes, proteins, metabolites, complexes, diseases, interactions). Recently, mappings to the EBI Complex Portal (27) and IUPHAR/BPS Guide to PHARMACOLOGY (28) have been added to the identifier mapping framework BridgeDb (29) which is integrated with WikiPathways.

### Wikimedia Toolforge

The pathway viewer widget was updated and moved to Wikimedia Toolforge allowing users to integrate the pathways from WikiPathways into their own website with more ease (http://widget.wikipathways.org). The widget unifies identifiers from the data source originally specified by the pathway author, to provide several commonly used data sources for additional exploration. Metabolite identifiers are unified by BridgeDb to include ChEBI, HMDB and Wikidata identifiers. For gene products, the Wikidata API is used for mapping of NCBI Gene, Ensembl, HGNC and Wikidata identifiers. In the future, the mapping for gene products will also be done using BridgeDb.

### SPARQL explorer

**Figure 4.**
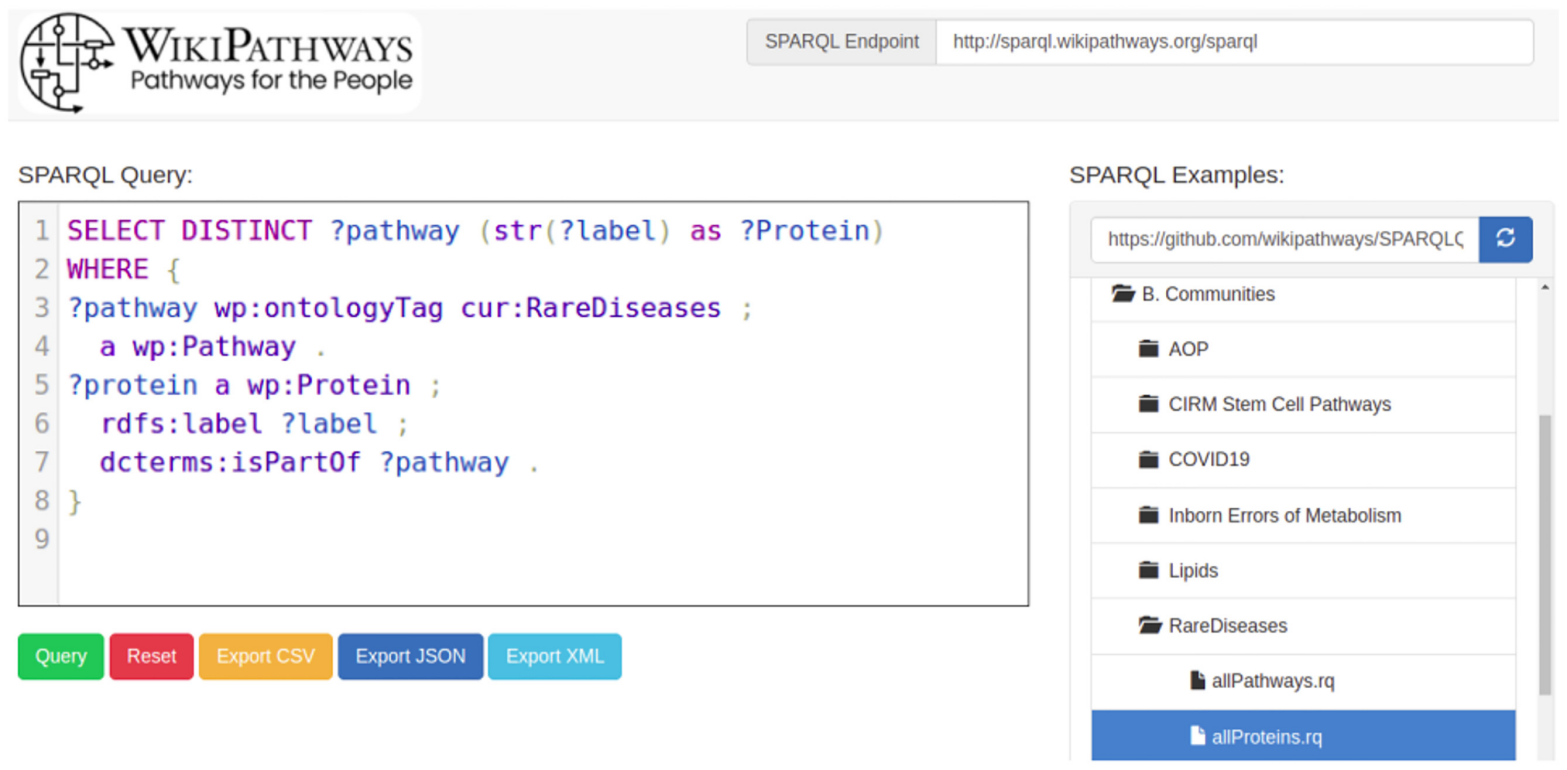
The WikiPathways SNORQL user interface (http://sparql.wikipathways.org). The WikiPathways SNORQL semantic web browser allows for user-friendly access to the WikiPathways RDF through providing example SPARQL queries (right panel).

To make access to the WikiPathways RDF more user-friendly, we have introduced Wikipathways SNORQL (https://github.com/wikipathways/snorql-extended), a new extended implementation of the SNORQL user interface (UI), to be our go-to semantic web browser (http://sparql.wikipathways.org). WikiPathways SNORQL is a query editor that offers syntax highlighting for writing and executing SPARQL queries directly on our existing SPARQL endpoint (http://sparql.wikipathways.org/sparql). Furthermore, the user interface provides a query examples panel which is auto-populated with SPARQL queries from a customizable GitHub repository (https://github.com/wikipathways/SPARQLQueries). This repository stores queries in folders divided over particular topics, communities, collaborations functionality, or external data sources for federated queries, allowing new users to navigate through our example queries panel with more ease (Figure 4). Overall, the new UI allows collecting, storing, exploring and reusing SPARQL queries on the WikiPathways data.

One example of semantic interoperability using federated SPARQL queries is our collaboration with neXtProt. The neXtProt (30) knowledge resource of the Swiss Institute of Bioinformatics aims to document inter- and intra-individual diversity of human proteins by integrating information from a variety of protein resources. To extend the knowledge on proteins towards systems and biological pathways, federated SPARQL queries have been developed in collaboration, harnessing the semantic web capabilities to connect neXtProt with WikiPathways knowledge.

### Wikidata

The CC0 license of WikiPathways also enabled interoperability through Wikidata, the linked data repository of Wikipedia (31). Like Wikipedia, Wikidata is a knowledge-sharing platform open to all (humans and software). In collaboration with the Gene Wiki and Reactome teams, we developed bots to add information about the curated pathways to Wikidata (19,32). The WikiPathways bot creates Wikidata items for each pathway and the content thereof in WikiPathways and aligns those with the Wikidata items on associated genes, proteins, metabolites, literature citations, and ontology annotations (e.g. https://www.wikidata.org/wiki/Q28031254). The WikiPathways content of the human pathway analysis collection in Wikidata gets updated after each monthly release.

### Scholia

Scholia is a graphical user interface that aggregates information from Wikidata around topics (33,34), such as genes, proteins, metabolites, pathways, authors, articles, and organizations. In collaboration with the Scholia team, we developed Scholia templates for WikiPathways pathways and all pathways in Wikidata are now also addressable as Scholia topic pages https://scholia.toolforge.org/wikipathways/WP111. These pages show the participants of the pathway (genes, protein, metabolites), the literature cited by the pathway, and articles citing the pathway. Moreover, similar to linking to PubMed and Europe PMC, the literature section now links to Scholia pages for the cited articles.

### Nanopub

Nanopublications for WikiPathways have been released for several years now in collaboration with their international community (35). The nanopublications are created using a combination of nanopub-java library (36) and SPARQL queries against the WikiPathways RDF (see https://github.com/wikipathways/nanopublications). This results in three types of nanopublications, for three types of facts in WikiPathways: complexes, interactions, and general participation in pathways. Nanopublications are currently only generated if the complex, interaction or participation is linked to a specific literature reference, identified by a PubMed identifier, which is used as part of the provenance of the nanopublication. Nanopublications are findable using semantic web identifiers for genes and proteins (37), but using the pathway identifier we can also find all nanopublications that originate from the corresponding pathway, such as WP15, for example with a command line:

curl -X GET ‘http://grlc.np.dumontierlab.

com/api/local/local/find_nanopubs_with_uri

?ref=http://identifiers.org/wikipathways/

WP15_r107118’ -H ‘accept: text/csv’

### Europe PMC and other PubMed interoperability

The PubMed identifier is still the primary, global identifier used by WikiPathways to identify the literature cited. In 2018 we started contributing links between PubMed articles and pathways in WikiPathways (excluding the Reactome-synced pathways (5)) to Europe PMC via the External links service (https://europepmc.org/LabsLink) functionality (38). This allows Europe PMC to show the pathways from WikiPathways that mention that article in their database. The WikiPathways website now also links to Europe PMC for cited articles in the literature section on a pathway page, making the integration bi-directional.

### Enrichment analysis tools

Functional enrichment analysis is a popular approach for characterizing differentially expressed genes based on Gene Ontology terms, pathways and other annotated gene sets. We release a standard Gene Matrix Transposed (GMT) file each month that includes the latest set of curated pathways approved for enrichment use cases. Using this file, WikiPathways content can be added to any protocol supporting the GMT standard. A number of R packages and online tools that perform enrichment analysis have incorporated WikiPathways into their methods and vignette examples, including g:Profiler (39), clusterProfiler (40), rSEA (41), Enrichr (42), IMPaLa (43) and WebGestalt (44). Human and mouse WikiPathways gene sets are now also available in the Molecular Signatures Database (MSigDb, (45)) for the GSEA software (46).

### MINERVA

The interoperability between the MINERVA platform (47) and the WikiPathways GPML got a boost with the COVID-19 Disease Map project (8). In this large international effort, it is crucial to be able to communicate between the different resources to share, collect and unify the content. The MINERVA software can now import and export GPML files from WikiPathways. This facilitates the integration of WikiPathways pathways in the larger disease map but also allows export of other models to GPML enabling distribution of the content in RDF format in the future.

### BEL ecosystem

Several researchers involved in the Biological Expression Language (BEL) project (48) are harmonizing the information of different pathway databases including WikiPathways. Bio2BEL converts the content from several pathway databases into BEL and stores causal and correlative relations between biological entities across multiple modes and scales as a biological network (49). ComPath aims to evaluate the coverage, agreements, and discrepancies between the pathway databases in terms of gene content (50). PathMe provides normalizations between these pathway databases for other content (e.g. protein–protein interactions, complexes) (51). This information is integrated into PathwayForte (52) and the feedback from these analyses led to additional curation efforts on WikiPathways including renaming of pathways.

### Network Data Exchange - NDEx

The Network Data Exchange (NDEx) is a public resource for publishing and sharing biological networks and gene sets (53), and WikiPathways is closely collaborating with the NDEx team. First, we regularly deposit the WikiPathways human pathway analysis collection into a dedicated collection at NDEx (http://ndexbio.org/#/user/363f49e0-4cf0-11e9-9f06-0ac135e8bacf), with over 600 pathways included. Second, as part of the Clinical Proteomic Tumor Analysis Consortium (CPTAC) initiative, we have led the curation of 28 cancer-specific pathway models and the organization of 87 cancer-related pathways, all of which we have merged into 11 network models representing common cancer hallmark categories (http://cptac.wikipathways.org). We regularly deposit these network models at NDEx. Third, we have extracted gene sets from over 32,000 pathway figures with 10 or more genes and have deposited them at NDEx (53) for manual download and programmatic access, as well as facilitated Cytoscape workflows and enrichment analysis via NDEx Integrated Query (http://iquery.ndexbio.org).

## FUTURE WORK

With the ever-growing collection of curated and published pathway information, the WikiPathways team is working towards a more sustainable and scalable infrastructure for all pathway knowledge. This work will involve the development of new tools and services, continued integration into community-run resources like Wikidata, and close coordination with other pathway databases and biocuration teams. For example, we are building a pathway knowledge management system using git version control, with automated diff and merge capabilities to synchronize curation efforts across multiple sites. Pathway edits made at Wikidata, NDEx, WikiPathways or Reactome would essentially open a request to merge and redistribute the new information. Utilizing gene, metabolite and disease content extracted from published pathway figures, we will organize focused curation efforts aimed at converting content as pathway models. This process will be facilitated by the addition of a portal highlighting and organizing this content.

We are also integrating pathway information into new platforms for biomedical research and discovery. This work includes the continued support for third-party pathway analysis tools online and via R and Python packages, as well as new knowledge bases that connect pathway information to other genomic and phenotypic models of biology. For example, we are contributing curated and published pathway information to the NCATS’ Biomedical Data Translator program (54) using the BioThingsExplorer (https://biothings-explorer.readthedocs.io) and Smart API (55).

## CONCLUSION

The content of WikiPathways increases every day due to the combined effort of multiple communities, including curators of pathway models and authors of pathway figures. A wide variety of third-party analytical tools utilizes the content distributed by WikiPathways, creating an immeasurable user base. WikiPathways is defined by the people authoring, curating and utilizing pathway knowledge. We invite all researchers interested in pathways to directly participate in the WikiPathways project.

## DATA AVAILABILITY

All WikiPathways data, including older data releases, are stored on data.wikipathways.org. Scripts and SPARQL queries used to generate the data published here can be found on GitHub (56).
